# Ciliary Neurotrophic Factor Derived From Astrocytes Protects Retinal Ganglion Cells Through PI3K/AKT, JAK/STAT, and MAPK/ERK Pathways

**DOI:** 10.1167/iovs.63.9.4

**Published:** 2022-08-04

**Authors:** Kwanghyun Lee, Jin-Ok Choi, Ahreum Hwang, Hyoung Won Bae, Chan Yun Kim

**Affiliations:** 1 Institute of Vision Research, Department of Ophthalmology, Yonsei University College of Medicine, Seoul, Republic of Korea; 2 Department of Ophthalmology, National Health Insurance Service Ilsan Hospital, Goyang, Gyeonggi-do, Republic of Korea

**Keywords:** astrocytes, retinal ganglion cells, neurotrophins, apoptosis, neuron-glia interactions

## Abstract

**Purpose:**

The purpose of this study was to investigate the roles of ciliary neurotrophic factor (CNTF) on the protective effects of astrocytes on retinal ganglion cells (RGCs).

**Methods:**

Primary RGCs were isolated from neonatal rats. Oxidative stress was induced, and the effects of co-culture with astrocytes and CNTF treatment on RGCs were evaluated. The pathways commonly altered by astrocytes and CNTF were investigated. Effects of each pathway were investigated using pathway inhibitors against PI3K/AKT, JAK/STAT, and MAPK/ERK. RNA sequencing was performed to identify the genes upregulated and downregulated by CNTF treatment.

**Results:**

Astrocytes improved the viability and increased β3-tubulin expression in RGCs. The concentration of CNTF increased in the RGC-astrocyte co-culture medium. The protective effects of astrocytes were abolished by neutralization with the anti-CNTF antibody; thus, CNTF may play an important role in the effects mediated by astrocytes. Furthermore, CNTF treatment alone enhanced the viability and β3-tubulin expression of RGCs and increased the population of viable RGCs under oxidative stress. The PI3K/AKT pathway was associated with both RGC viability and β3-tubulin expression. However, the JAK/STAT pathway increased the viability of RGCs, whereas the MAPK/ERK pathway was associated with β3-tubulin expression. RNA sequencing revealed the CNTF-upregulated genes associated with response to DNA damage and downregulated genes associated with photoreceptor cell differentiation.

**Conclusions:**

Our data revealed protective effects of astrocyte-derived CNTF on RGCs. In addition, we showed that multiple pathways exert these protective effects and identified the novel genes involved. These results may be helpful in developing treatments for RGC injury.

Retinal ganglion cells (RGCs) are located in the innermost layer of the retina and are the last cells of the neural network that receive visual stimuli within the eyes. Their axons form the optic nerve, which transmits visual information to the brain.^1^ Therefore, RGCs play a crucial role in vision, and their loss can result in irreversible blindness, as seen in ocular diseases such as glaucoma.^2^ RGC death occurs as a result of increased intraocular pressure, advanced glycation end products, or mitochondrial dysfunction,^3^^–^^5^ and once RGCs are damaged by these causes, they do not recover spontaneously like other neurons in the central nervous system (CNS).^4^^,^^6^^–^^10^

Astrocytes are involved in various critical functions for normal neuronal activity, including glutamate uptake, glutamine release, K^+^ and H^+^ buffering, water transport, and metabolic and nutritional support.^11^ However, in response to neuronal injury and neuroinflammation, astrocytes are activated and may worsen neuronal injury by releasing glutamate, secreting nitric oxide and pro-inflammatory factors, and forming glial scars.^7^^,^^12^ Therefore, astrocytes have long been considered to be associated with the failure of axon regeneration, and many studies have attempted to eradicate or restrict astrocytes to facilitate axon regeneration following a CNS injury.^12^^–^^14^ Recent studies, however, have indicated that astrocytes are required for successful neuronal survival in the CNS.^4^^,^^9^^,^^12^^,^^15^^–^^17^ Therefore, achieving a proper balance, rather than completely suppressing the astrocytes, may be critical for appropriate neuronal repair.

The purpose of this study was to clarify the effects of astrocytes on RGCs, especially under oxidative stress. We examined the impact of astrocytes on the viability and β3-tubulin (a marker of neurons in the central and peripheral nervous systems^18^) expression of RGCs. In addition, we investigated the factors that contribute to these effects and the pathways that are involved in this process. The findings of this study may be useful for the development of neurodegenerative disease treatments involving modulating astrocytes or astrocyte-derived factors.

## Methods

### Animals

A total of 42 pregnant Sprague-Dawley rats were purchased from Orientbio Inc. (Seongnam, Republic of Korea). Newborn rat pups (*n* = 360) were euthanized by decapitation. The study was approved by the Institutional Animal Care and Use Committee of Yonsei University College of Medicine, Seoul, Republic of Korea (2019-0150), and the rats were treated according to the Association for Research in Vision and Ophthalmology Statement for the Use of Animals in Ophthalmic and Vision Research. Substantial effort was made to minimize the number of animals euthanized and their suffering.

### Preparation of RGCs

RGCs were harvested from 2- or 3-day-old newborn rats as previously described, using a 2-step immunopanning method.^19^ Briefly, the retinal tissue was separated from the enucleated eyeballs, and the mixed retinal cells were collected as a suspension. The retinal cell suspension was incubated with rabbit anti-rat macrophage antibody (1:50 dilution; Fitzgerald Industries International, Acton, MA, USA) for 5 minutes. The suspension was placed in a 100-mm Petri dish coated with goat anti-rabbit immunoglobulin G antibody (1:200 dilution; Southern Biotechnology Associates, Birmingham, AL, USA) for 30 minutes. Non-adherent cells were then placed in a second 100-mm Petri dish coated with mouse anti-rat thymocyte differentiation antigen (Thy) 1.1 antibody (1:50 dilution; Bio-Rad, Hercules, CA, USA) for 1 hour and subsequently incubated with anti-biotin magnetic MicroBeads (Miltenyi Biotec*,* Bergisch Gladbach, Germany). Finally, the magnetic-labeled RGCs were collected using a magnetic separating unit. All procedures were conducted at room temperature in a laminar flow hood. The isolated cells were cultured in Dulbecco's modified Eagle's medium/nutrient mixture F-12 (DMEM/F-12; Catalog no. SH30023.01; HyClone Laboratories, Logan, UT, USA) containing 10% fetal bovine serum (FBS; Life Technologies, Grand Island, NY, USA), 100 U/mL penicillin, and 100 µg/mL streptomycin (Life Technologies). Cells were seeded onto 12-mm glass coverslips precoated with poly-L-ornithine and laminin (Sigma-Aldrich, St. Louis, MO, USA). The cultures were incubated at 37°C in humidified 5% CO_2_ and 95% air. The cultured RGCs were validated by BRN3 (1:1,000, SC-8429, Santa Cruz Biotechnology, Santa Cruz, CA, USA) immunostaining (Supplementary Fig. S1).

### Preparation of Astrocytes and Müller Cells

Primary rat optic nerve head astrocytes were isolated from optic nerve heads of Sprague-Dawley rats according to a protocol modified from previous studies.^20^ Briefly, rat pups were euthanized by decapitation at postnatal day 0 to 1. One litter of pups (15 animals) was used for each cell culture experiment. Optic nerve head tissue was dissected proximal to the sclera and was digested for 15 minutes using 0.25% trypsin (Invitrogen, Carlsbad, CA, USA) at 37°C. Cells were washed once with optic nerve head astrocyte growth medium (DMEM/F-12 with 10% FBS, 100 U/mL penicillin, and 100 µg/mL streptomycin), and spun for 5 minutes at 1500 revolutions per minute (rpm). The cells were resuspended in optic nerve head astrocyte growth medium and cultured in laminin-coated T75 cell culture flasks. Then, the cells were maintained in a humidified incubator containing 5% CO_2_ at 37°C. The medium was left unchanged for 3 days, and the cultured cells were then used for the experiments. The cultured astrocytes were validated by GFAP (G3893, Sigma; 1:1,000) immunostaining (Supplementary Fig. S2).

Müller cells were isolated by the expansion culture method.^21^^–^^23^ Dissociated retinal cells from 15 rats were plated onto laminin-coated culture dishes containing DMEM/F-12, 10% FBS, 100 U/mL penicillin, and 100 µg/mL streptomycin. The medium was left unchanged for 5 days and subsequently replaced every 3 days.

### Co-Culture System

RGCs and astrocytes (each seeded at 2 × 10^5^/well) were co-cultured in the same well without direct contact. To prepare the co-cultures of RGCs and confluent astrocytes, passaged astrocytes were precultured onto a nitrocellulose membrane (membrane area, 0.6 cm^2^; Millicell*-*CM; Millipore, Bedford, MA, USA) in 24-well plates 3 to 4 days prior to co-culture and cultured in medium (DMEM/F-12, 10% FBS, 100 U/mL penicillin, and 100 µg/mL streptomycin) until 70% to 80% confluence was reached. RGCs were seeded into the bottoms of wells in 24-well plates. Twenty-four hours after RGC seeding, membranes containing confluent astrocytes were placed over the seeded RGCs in the wells. Co-cultures were incubated for 24 hours. RGCs that were cultured beneath a blank nitrocellulose membrane insert were used as the control. For neutralization, 50 µg/mL anti-rat CNTF antibody (R & D systems, Minneapolis, MN, USA) was applied to the culture medium immediately before membrane insertion.

### Oxidative Stress and CNTF Treatment

At 24 hours after seeding, RGCs were stressed by the addition of 25 µM hydrogen peroxide (H_2_O_2_; Sigma-Aldrich, St. Louis, MO, USA) for 24 hours. The concentration of H_2_O_2_ was based on the concentration required to reduce the number of the surviving RGCs by 50% when oxidative stress was applied (Supplementary Fig. S3). Recombinant rat CNTF (PeproTech, London, UK) was added at a concentration of 0 to 100 ng/mL for 24 hours. After that, CNTF was treated with RGCs for 24 hours at a concentration of 50 ng/mL, which was thought to be the concentration for maximum effect. To assess the role of each signal transduction pathway, pathway inhibitors were administered in the same environment, including the PI3K/AKT pathway inhibitor LY294002 (50 µM; Cell Signaling Technology), MAPK/ERK pathway inhibitor PD98059 (50 µM; Cell Signaling Technology), or JAK/STAT3 pathway inhibitor AG490 (10 µM; Cell Signaling Technology), 4 hours before recombinant CNTF treatment, at concentrations reported in previous studies.^24^ RNA sequencing was performed under the same conditions without oxidative stress.

### Cell Viability

The viability of cultured RGCs was determined by quantification of the ATP levels, which indicate the presence of metabolically active cells (CellTiter-Glo Luminescent Cell Viability Assay; Promega, Madison, WI, USA).^25^ For each well in the opaque-walled 96-well plate, a volume of CellTiter-Glo Reagent (100 µL) was added to the same volume of cell culture medium (100 µL) containing the cells. To induce cell lysis, the contents were mixed for 2 minutes in an orbital shaker. The solution was allowed to stabilize for 10 minutes at room temperature, and the luminescent signal was recorded.

### Western Blotting

Total cell lysates were obtained using cell lysis buffer (Cell Signaling Technology) and incubated on ice for 5 minutes. The lysates were then sonicated, and the cell homogenates were centrifuged at 15,000 × *g* for 15 minutes at 4°C. Next, the concentration of proteins in the supernatants was measured using the Pierce Bicinchoninic Acid Protein Assay Kit (Thermo Fisher Scientific). Soluble proteins (3 µg per sample) were boiled for 5 minutes and resolved using 10% sodium dodecyl sulfate-polyacrylamide gel electrophoresis. Proteins were then electrotransferred to polyvinylidene fluoride membranes (0.45 µm pore size) and blocked using 5% skim milk in Tris-buffered saline, 0.1% Tween 20 (TBS-T). Membranes were blotted overnight with the primary antibodies, namely anti-CNTF (Santa Cruz, Sc-365210), anti β3-tubulin (R&D Systems, Minneapolis, MN, USA), and anti-α-tubulin (Cell Signaling Technology) antibodies, diluted in 0.1% bovine serum albumin and 0.01% sodium azide in TBS-T. After washing 3 times with TBS-T, blots were incubated with horseradish peroxidase-conjugated secondary antibody (Cell Signaling Technology) for 1 hour at room temperature. Blots were washed three times with TBS-T, and immunoreactive bands were visualized through enhanced chemiluminescence. Relative intensities of immunoreactive bands were measured after normalization for α-tubulin levels.

### Flow Cytometry

Apoptosis was detected using an Annexin V-fluorescein thiocyanate (FITC) Apoptosis kit (BioVision, Milpitas, CA, USA), according to the manufacturer's directions. Briefly, following collagenase digestion, the isolated cells were stained with Annexin V-FITC and propidium iodide (BioVision) and analyzed using FACS LSRII (Beckman Coulter, Brea, CA, USA). The percentage of viable cells was determined as the percentage of Annexin V(+)/PI(+) cells in the samples.

### Enzyme-Linked Immunosorbent Assay 

The concentration of CNTF in the media was measured using a commercially available enzyme-linked immunosorbent assay (ELISA) kit specific for rat CNTF (RayBiotech Inc., Norcross, GA, USA) and was performed according to the manufacturer's instructions. Total protein concentrations were determined using the Pierce BCA Protein Assay Kit (Thermo Fisher Scientific, Inc., Rockford, IL, USA) as the standard.

### Quantitative Reverse-Transcription Polymerase Chain Reaction 

The RNeasy Micro Kit (Qiagen, Hilden, Germany) was used to isolate RNA, which was reverse-transcribed to cDNA using EcoDry Premix (Takara Bio, Mountain View, CA, USA). Real-time PCR was performed using SYBR Premix Ex Taq (Takara Bio) and pre-made primers. Reverse-transcription quantitative PCR (RT-qPCR) was performed on the StepOnePlus Real-Time PCR System (Applied Biosystems/Thermo Fisher Scientific, Foster City, CA, USA). Table 1 shows the sequences of the primers used. The results were analyzed using the comparative cycle threshold (C_T_) method and normalized to those of β-actin, which was used as an internal control in the same sample.

**Table 1.** Primers Used for Quantitative Reverse-Transcription Polymerase Chain Reaction

Target Gene	Sequence
*CNTF*	F: 5ʹ–CAC CCC AAC TGA AGG TGA CT–3ʹ R: 5ʹ–ACC TTC AAG CCC CAT AGC TT–3ʹ
*VEGF*	F: 5ʹ–GGC TCT GAA ACC ATG AAC TTT CT–3ʹ R: 5ʹ–GCA GTA GCT GCG CTG GTA GAC–3ʹ
β-Actin	F: 5ʹ–CAC CCG CGA GTA CAA CCT T–3ʹ R: 5ʹ–CCC ATA CCC ACC ATC ACA CC–3ʹ

CNTF, ciliary neurotrophic factor; VEGF, vascular endothelial growth factor; F, forward; R, reverse.

### Microarray

For pathway analysis between RGCs versus RGCs with astrocytes and RGCs versus RGCs with CNTF treatment, microarray analysis was performed. In addition, the genes, whose expression was altered in astrocytes when cultured with RGCs, was also analyzed. RNA extraction and the following RNA microarray and Kyoto Encyclopedia of Genes and Genomes (KEGG) pathway analysis were performed by Macrogen Inc. (Seoul, Korea). Briefly, cDNA was synthesized using the GeneChip Whole Transcript (WT) amplification kit according to the manufacturer's instructions. Thereafter, the sense cDNA was fragmented and biotin-labeled with TdT using the GeneChip WT Terminal labeling kit. A 5.5-µg sample of labeled DNA target was hybridized for 16 hours at 45°C on the GeneChip Rat Gene 2.0 ST Array (Affymetrix). The GeneChips were washed and stained in the Fluidics Station 450 (Affymetrix) and then scanned by using the GeneChip Scanner 3000 (Affymetrix). The probe cell intensity data computation and a CEL file generation was performed using Affymetrix GeneChip Command Console Software (AGCC). The data were summarized and normalized with the robust multi-average (RMA) method implemented in Affymetrix Power Tools (APT). We exported the result with gene-level RMA analysis and performed the differentially expressed gene (DEG) analysis. Statistical significance of the expression data was determined using fold change. Fold change filters were set to retain only upregulated genes that were greater than or equal to two-fold of the controls and downregulated genes present in less than or equal to half-fold of the controls. Gene enrichment and functional annotation analysis for the significant gene list was performed using Gene Ontology (http://geneontology.org) and KEGG (http://kegg.jp). All data analysis and visualization of DEGs were conducted using R version 3.3.2 (www.r-project.org).

### RNA Sequencing

Preparation of the RNA libraries and sequencing were performed by LAS Inc. (Gimpo, Korea; http://www.lascience.co.kr/). The libraries were prepared using the SMARTER Stranded Total RNA-seq kit-v2–Pico input mammalian (Pico) by Takara Bio according to the manufacturer's protocol. The RNAs were briefly ligated with 3′ and 5′ adaptors and reverse-transcribed to cDNA. PCR was then performed using different Illumina index primers, for distinguishing multiple timepoints after injury in the proximal and distal segments. All libraries were sequenced on the NextSeq 500 System (Illumina, San Diego, CA, USA), with 75-bp paired-end reads.

Read-quality check was performed using FastQC version 0.11.5.^26^ Sequencing adapters and low-quality bases in the raw reads were trimmed using Skewer version 0.2.2.^27^ The cleaned high-quality reads after trimming the low-quality bases and sequencing adapters were mapped to the reference genome using STAR version 2.6 software.^28^

To quantify the mapped reads onto the reference genome into the gene expression values, Cuffquant in Cufflinks version 2.2.1 was used.^29^ The gene annotation of the reference genome rn6 from the UCSC genome (https://genome.ucsc.edu) in GTF format was used for gene models, and the expression values were calculated as fragments per kilobase of transcript per million mapped reads. The DEGs between the two selected biological conditions (RGCs versus RGCs with CNTF treatment) were analyzed using Cuffdiff in the Cufflinks package.^30^ Genes with a fold change cutoff of 2 and a *P* value cutoff of 0.05 were considered differentially expressed. To compare the expression profiles among the samples, the normalized expression values of the selected few hundred of the DEGs were unsupervised clustered using R scripts written by LAS Inc. The scatter plots for the gene expression values and volcano plots for the expression fold changes and *P* values between the two selected samples were also drawn.

To obtain insight on the biological functional role of the DEGs, overlapping tests for gene sets between the analyzed DEGs and functionally categorized genes, including biological processes of Gene Ontology, KEGG pathways, and other functional gene sets, were run using g:Profiler2 version 0.2.0.^31^

### Statistical Analysis

Each experiment was conducted in triplicate, and all data are expressed as the mean ± standard deviation (SD). Differences between groups were examined using Student's *t*-test and 1-way analysis of variance (ANOVA and subsequent post hoc analysis with Dunnett's test). All statistical analysis were performed using GraphPad Prism version 9.0 for Windows (GraphPad Software, San Diego, CA, USA). A *P* value <0.05 was considered statistically significant.

## Results

### Protective Effects of Astrocytes on RGCs

**Figure 1.**
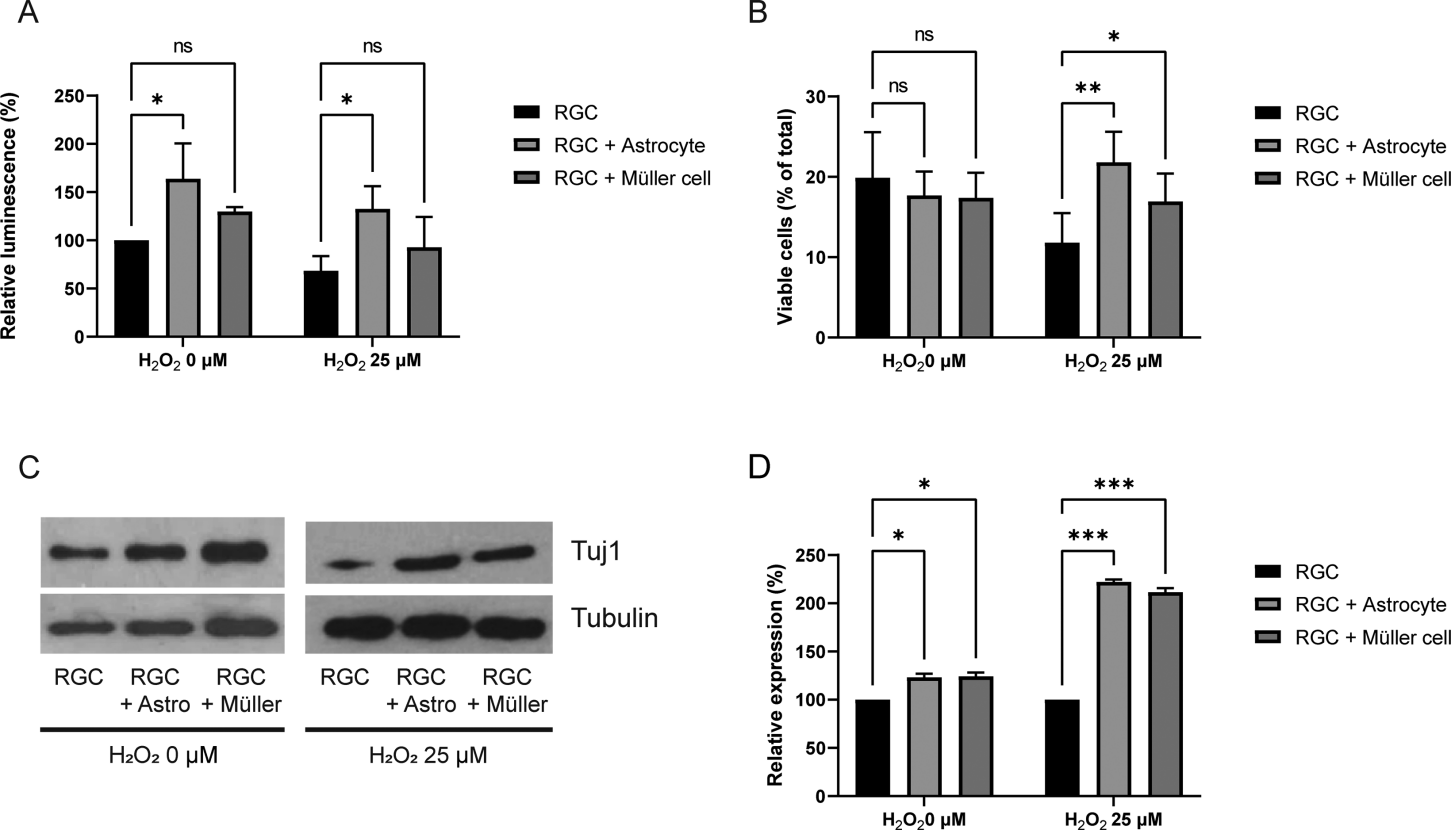
Effects of astrocytes and Müller cells on retinal ganglion cells (RGCs). RGCs and glial cells were co-cultured in the same well without direct contact, with a membrane between them. An ATP assay was used to determine viability, and flow cytometry to determine the percentage of viable RGCs, and Western blotting to detect β3-tubulin expression. (**A**) RGC viability was increased when co-cultured with astrocytes with and without oxidative stress induced by H_2_O_2_. However, the effects of Müller cells were not significant. (**B**) Flow cytometry results. When RGCs were co-cultured with astrocytes or Müller cells under oxidative stress, the percentage of viable cells were increased. (**C**) β3-tubulin (Tuj 1) expression was increased when RGCs were cultured with astrocytes with and without oxidative stress. (**D**) Quantification of Western blot bands described in **C**. Both astrocytes and Müller cells increased the expression of β3-tubulin without and with oxidative stress. *, *P* < 0.05; **, *P* < 0.01; ***, *P* < 0.001. Data in the columns indicate the mean ± SD of three experiments. Error bars were not displayed when RGCs were cultured alone in the ATP assay without oxidative stress and when RGCs were cultured alone in the Western blot (with or without oxidative stress), as they were set as the standard in the experiment.

To evaluate the effect of glial cells, RGCs were co-cultured with astrocytes or Müller cells for 24 hours, and their viability was compared to that of RGCs cultured alone. The RGCs cultured with astrocytes showed increased viability compared to the RGCs cultured alone. When compared to the control (RGCs alone), the viability of RGCs cultured with astrocytes without oxidative stress was increased to 164.0 ± 36.5% (*P* = 0.017). Oxidative stress decreased the viability of RGCs cultured alone to 68.5 ± 15.2% (*P* = 0.023). The viability of the RGC-astrocyte culture, under oxidative stress, was increased to 132.7 ± 23.4% (*P* = 0.016; Fig. 1A). According to flow cytometry, the co-culture did not significantly alter the percentage of viable cells without oxidative stress. However, under oxidative stress, the percentage of viable cells was greater when cultured with either astrocytes (21.8 ± 3.8%, *P* = 0.009) or Müller cells (17.0 ± 3.5%, *P* = 0.025) than when RGCs were cultured alone (11.8 ± 3.7%). Compared to Müller cells, astrocytes had a stronger influence on RGCs (Fig. 1B). β3-tubulin expression in RGCs was also increased by co-culture with astrocytes without and with oxidative stress (*P* = 0.014 and *P* < 0.001, respectively), as well as co-culture with Müller cells (*P* = 0.015 and *P* < 0.001, respectively; Figs. 1C, 1D) without and with oxidative stress.

### CNTF Synthesis by Astrocytes

Using microarray, we compared the degree of change in the expression of neurotrophic factor genes in astrocytes cultured with RGCs to that in astrocytes cultured alone. The expression of the *CNTF* gene in astrocytes cultured with RGCs was increased by 2.765-fold compared with astrocytes cultured alone (Table 2). However, the expression of other genes, including brain-derived neurotrophic factor (*BDNF*), glial cell-derived neurotrophic factor (*GDNF*), fibroblast growth factor 2 (*FGF2*), nerve growth factor (*NGF*), and neurotrophin-3 (*NT-3*), were not significantly changed (fold change: 1.012, 1.120, 1.070, 1.108, and 0.901, respectively). In the case of vascular endothelial growth factor (*VEGF*), a difference in expression was observed depending on the gene type: *VEGFA* had a fold change of 2.171, *VEGFB* had a fold change of 0.810, and *VEGFC* had a fold change of 1.236 (see Table 2).

**Table 2.** Microarray Analyzing the Transcriptome in Astrocytes With or Without Co-Culture With RGCs

Gene Symbol	Astrocytes Alone (Log2)	Astrocytes + RGCs (Log2)	Fold Change
*BDNF*	5.349990	5.366730	1.011671
*CNTF*	4.521250	5.988590	2.765116
*GDNF*	5.467380	5.630770	1.119916
*NGF*	6.943920	7.091960	1.108063
*NT-3*	3.074350	2.923340	0.900620
*VEGF A*	7.421940	8.540300	2.171000
*VEGF B*	9.217950	8.914310	0.810206
*VEGF C*	8.057370	8.362510	1.235539
*FGF2*	6.530990	6.628290	1.069770

RGCs, retinal ganglion cells; BDNF, brain-derived neurotrophic factor; CNTF, ciliary neurotrophic factor; GDNF, glial cell line-derived neurotrophic factor; NGF, nerve growth factor; NT-3, Neurotrophin-3; VEGF, vascular endothelial growth factor; FGF, fibroblast growth factor.

**Figure 2.**
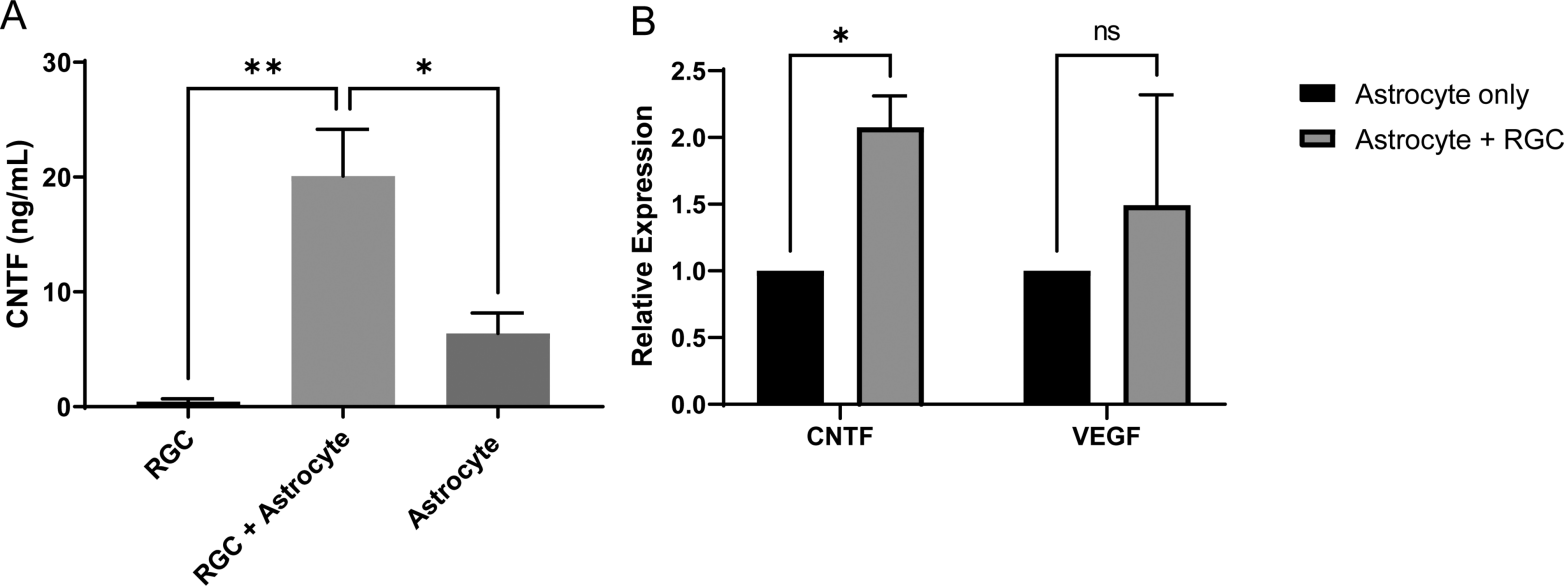
Increased synthesis of ciliary neurotrophic factor (CNTF) in astrocytes. (**A**) Enzyme-linked immunosorbent assay (ELISA) analysis of CNTF expression in media. When retinal ganglion cells (RGCs) were cultured with astrocytes, the concentration of CNTF in the media increased significantly. (**B**) Real-time PCR analysis of mRNA expression in astrocytes cultured alone or with RGCs. RT-PCR results showed that transcription of CNTF was increased in astrocytes when astrocytes were cultured with RGCs. The transcription of vascular endothelial growth factor (*VEGF*) was not significantly altered. Data in the columns indicate the mean ± standard deviation (SD) of three experiments. *, *P* < 0.05; ns, not significant. Error bars were not displayed when astrocytes were cultured alone, as they were set as the standard in the experiment.

The increased concentration of CNTF in culture media was also confirmed using ELISA. In the culture medium containing only RGCs, a very low concentration of CNTF, 0.30 ± 0.25 ng/mL, was detected. However, when RGCs were co-cultured with astrocytes, the level of CNTF in the medium increased significantly to 17.25 ± 6.67 ng/mL (*P* = 0.005), which was also greater than that of only astrocytes at 6.38 ± 1.78 ng/mL (*P* = 0.017; Fig. 2A). RT-PCR results revealed that *Cntf* mRNA expression was significantly increased in astrocytes co-cultured with RGCs (*P* = 0.024; Fig. 2B). In contrast, the co-culture had no significant effect on the synthesis of VEGF (*P* = 0.287; see Fig. 2B).

### Decreased Protective Effects of Astrocytes by Anti-CNTF Antibody

**Figure 3.**
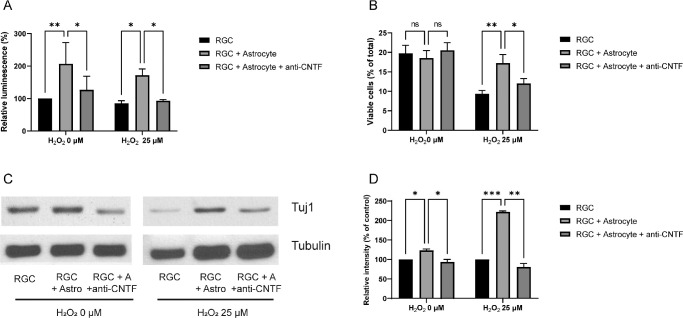
Critical role of ciliary neurotrophic factor (CNTF) in the protective effects of astrocytes. Anti-CNTF antibody inhibits the protective effects of astrocytes. Anti-CNTF (50 µg/mL) was added to the culture medium immediately before membrane insertion. (**A**) Anti-CNTF antibody treatment significantly decreased the viability of retinal ganglion cells (RGCs) co-cultured with astrocytes. (**B**) Flow cytometry results. Under oxidative stress, the percentage of viable RGCs increased by astrocyte co-culture was decreased by anti-CNTF treatment. (**C**) Western blots showing decreased β3-tubulin (Tuj 1) expression by anti-CNTF antibody. (**D**) Quantification of Western blot bands described in **C**. The expression levels of β3-tubulin relative to control were significantly decreased by anti-CNTF antibody treatment. Data in the columns indicate the mean ± SD of three experiments. *, *P* < 0.05; **, *P* < 0.01; ***, *P* < 0.001. Error bars were not displayed when RGCs were cultured alone in the ATP assay without oxidative stress and when RGCs were cultured alone in the Western blot (with or without oxidative stress), as they were set as the standard in the experiment.

For neutralization, the culture medium of the RGC-astrocyte culture was supplemented with 50 µg/mL anti-CNTF antibody; this decreased the viability of RGCs (see Fig. 3A). In the absence of oxidative stress, the viability of RGCs co-cultured with astrocytes was significantly decreased from 206.9% ± 65.3% to 127.2% ± 41.4% by anti-CNTF treatment (*P* = 0.023; see Fig. 3A). When oxidative stress was applied, the viability of RGCs with astrocyte co-culture decreased from 171.7% ± 19.4% to 93.5% ± 3.8% by anti-CNTF treatment (*P* = 0.025, Fig. 3A). According to flow cytometry results, anti-CNTF decreased the percentage of viable cells from 17.3 ± 2.2% to 12.0 ± 1.3% (*P* = 0.044; Fig. 3B). Anti-CNTF treatment also reduced the protein levels of β3-tubulin both with and without oxidative stress (*P* = 0.014 and *P* = 0.025, respectively; Figs. 3C, 3D).

### Effects of Exogenous CNTF on RGC Viability and β3-Tubulin Expression

**Figure 4.**
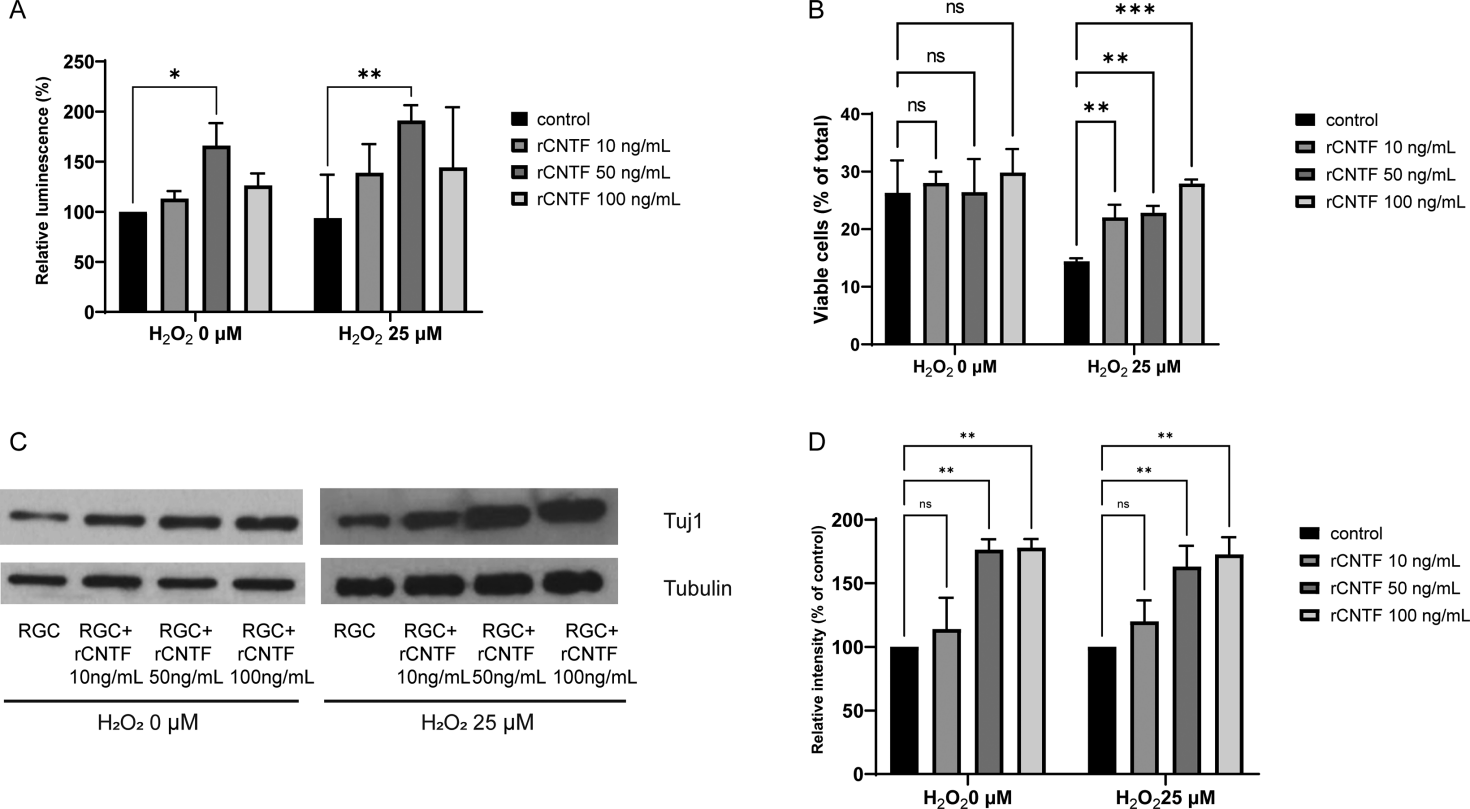
The effects of Ciliary neurotrophic factor (CNTF) on retinal ganglion cells (RGCs). Recombinant rat CNTF (rCNTF) at concentrations of 0 to 100 ng/mL was added 24 hours after seeding, and its effects were evaluated. (**A**) The viability of RGCs increased at concentrations up to 50 ng/mL CNTF but decreased at 100 ng/mL CNTF. (**B**) Flow cytometry results. Under oxidative stress, the percentage of viable RGCs was increased, as the concentration of rCNTF increased. (**C**) β3-tubulin (Tuj 1) expression due to rCNTF, evaluated using western blotting. (**D**) Quantification of Western blot bands described in **C**. β3-tubulin expression was prominent at the concentration of 50 ng/mL and 100 ng/mL CNTF compared to that in the control. Data in the columns indicate the mean ± standard deviation (SD) of three experiments. *, *P* < 0.05; **, *P* < 0.01; ***, *P* < 0.001. Error bars were not displayed when RGCs were cultured alone in the ATP assay without oxidative stress and when RGCs were cultured alone in the Western blot (with or without oxidative stress), as they were set as the standard in the experiment.

Figure 4A shows the increased viability of RGC when treated with 50 ng/mL of CNTF for 24 hours. Without oxidative stress, the measurements of RGC viability were 113.1% ± 7.6%, 166.0% ± 22.5%, and 126.3% ± 12.0% when incubated with 10, 50, and 100 ng/mL CNTF, respectively; the cell viability was considered 100% at a CNTF concentration of 0 ng/mL. Under oxidative stress, the viability of RGCs was 93.7% ± 43.2%, 139.0% ± 28.5%, 191.0% ± 15.4%, and 144.2% ± 60.0% when incubated with 0, 10, 50, and 100 ng/mL CNTF, respectively. At 50 ng/mL CNTF, the viability of RGCs was significantly improved without and with oxidative stress (*P* = 0.042 and *P* = 0.003, respectively). However, this improvement in the viability of RGCs without and with oxidative stress was not detected at 10 (*P* = 0.911 and *P* = 0.196, respectively) and 100 ng/mL CNTF (*P* = 0.587 and *P* = 0.135, respectively). In the absence of oxidative stress, CNTF did not alter the percentage of viable RGCs (Fig. 4B). However, the percentage of viable RGCs increased with the increase in CNTF concentration under oxidative stress. At 10, 50, and 100 ng/mL CNTF, the percentages of viable RGCs under oxidative stress were 22.1 ± 0.6% (*P* = 0.009), 22.9 ± 1.2% (*P* = 0.006), and 27.9 ± 0.7% (*P* < 0.001; see Fig. 4B), respectively, and were higher than that of RGCs cultured alone (14.4 ± 0.6%). The results of Western blotting revealed that without and with oxidative stress, the expression of β3-tubulin was significantly increased at 50 (*P* = 0.003 and *P* = 0.008, respectively) and 100 ng/mL (*P* = 0.003 and *P* = 0.004, respectively) CNTF (Figs. 4C, 4D).

### Roles of the JAK/STAT, PI3K/AKT, and MAPK/ERK Pathways in the Effects of CNTF on RGCs

**Figure 5.**
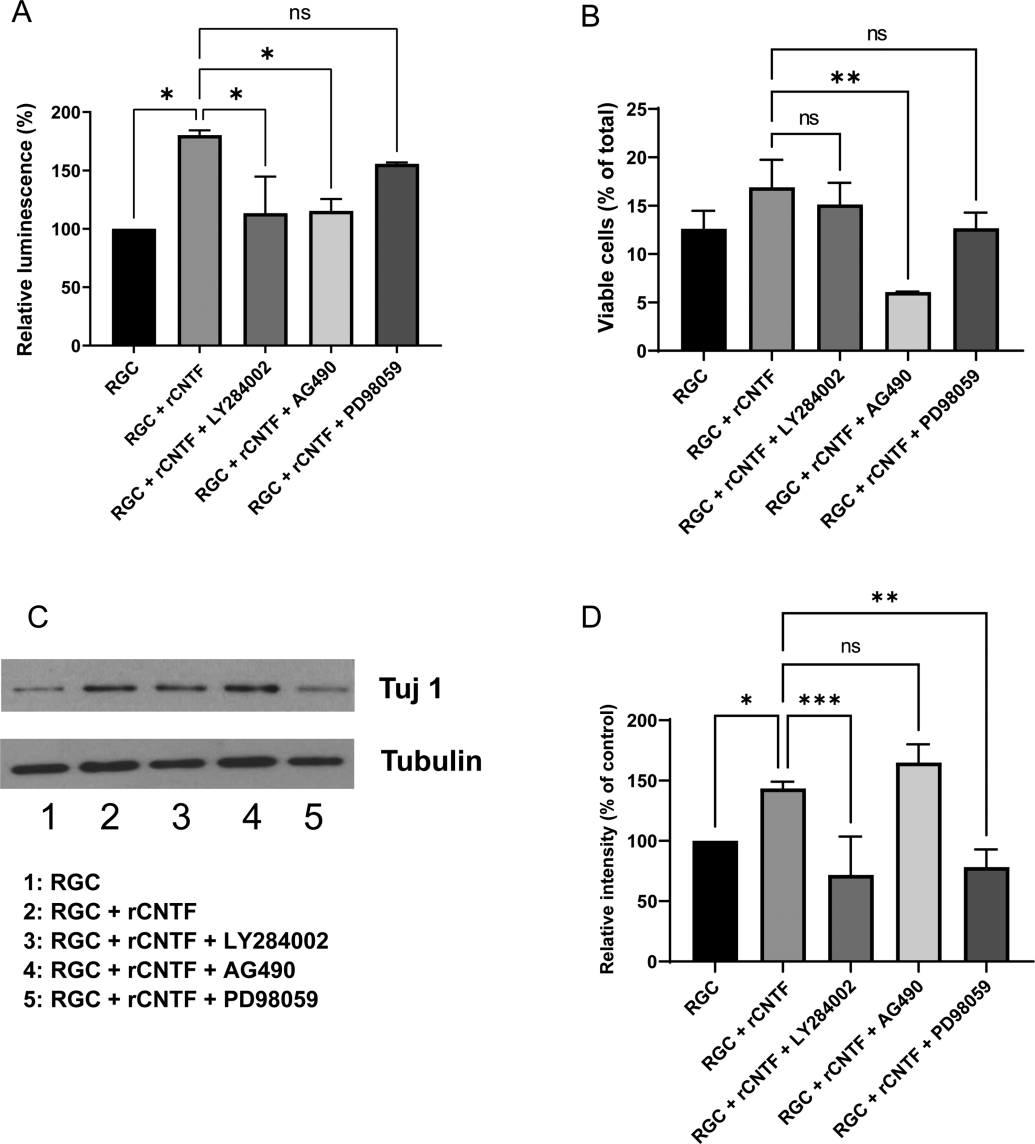
Role of PI3K/AKT, JAK/STAT and MAPK/ERK pathways in the effect of ciliary neurotrophic factor (CNTF) on retinal ganglion cells (RGC). CNTF enhances the viability of RGCs via the PI3K/AKT and JAK/STAT pathways, whereas β3-tubulin expression is increased via PI3K/AKT and MAPK/ERK pathways. RGCs were treated with pathway inhibitors, including the PI3K/AKT pathway inhibitor LY294002 (50 µM), MAPK/ERK pathway inhibitor PD98059 (50 µM), and JAK/STAT3 pathway inhibitor AG490 (10 µM), along with CNTF treatment. (**A**) Quantification of the viability of RGCs after treatment with LY294002, AG490, or PD98059 together with CNTF. The CNTF-enhanced viability of RGCs was decreased by LY284002 and AG490 but not by PD98059. (**B**) Flow cytometry results evaluating viable RGCs under oxidative stress. The viability of cells was increased by rCNTF but was significantly decreased after AG490 treatment. (**C**) Western blotting was performed to evaluate β3-tubulin (Tuj 1) expression of RGCs after treatment with LY294002, AG490, or PD98059 together with CNTF. (**D**) Quantification of Western blot bands shown in **C**. The expression levels of β3-tubulin relative to the control were significantly decreased by LY284002 and PD98059 but not by AG490. Data in the columns indicate the mean ± SD of three experiments. *, *P* < 0.05; **, *P* < 0.01; ***, *P* < 0.001; ns, not significant. Error bars were not displayed when RGCs were cultured alone in the ATP assay and when RGCs were cultured alone in the Western blot, as they were set as the standard in the experiment.

Microarray analysis was performed to uncover pathways involved in the effects of astrocytes and CNTF. Table 3 summarizes the pathways upregulated in RGCs treated with CNTF (50 ng/mL) compared to RGCs cultured alone. The pathways upregulated in RGCs cultured with astrocytes compared to RGCs cultured alone are also shown in the same table. Among signal transduction pathways, the MAPK/ERK, JAK/STAT, and PI3K/AKT pathways were significantly upregulated. To assess each pathway's functional effects, pathway inhibitors, including JAK/STAT pathway inhibitor (AG490), PI3K/AKT pathway inhibitor (LY294002), and MAPK/ERK pathway inhibitor (PD98059) were added to CNTF-treated RGCs. The JAK/STAT and PI3K/AKT pathway inhibition significantly decreased the viability of RGCs from 180.1% ± 4.2% to 115.2% ± 10.3% (*P* = 0.037) and 113.2% ± 31.4% (*P* = 0.033), respectively; however, the decrease in RGC viability by the MAPK/ERK pathway inhibitor was not significant, reaching 155.6% ± 1.2% (*P* = 0.535; Fig. 5A). According to the results of flow cytometry, only AG490 significantly decreased the percentage of viable cells when treated to CNTF-treated RGCs (*P* = 0.008; Fig. 5B). The results of Western blotting for β3-tubulin indicated that the PI3K/AKT and MAPK/ERK pathways played a significant role in mediating the effect of CNTF on RGCs whereas the JAK/STAT pathway did not (*P* = 0.001, *P* = 0.002, and *P* = 0.353, respectively; Figs. 5C, 5D).

**Table 3.** List of Enriched KEGG Pathways in RGCs Co-Cultured With Astrocytes and RGCs Treated With CNTF

	RGCs + Astrocytes		RGCs + CNTF Treatment	
KEGG Pathway	*P* Value	FDR	*P* Value	FDR
Olfactory transduction	0.008482852	0.028099448	3.94478E-08	9.74362E-06
Hepatitis C	0.001805703	0.011041232	5.89496E-07	4.85352E-05
Metabolic pathways	2.72084E-12	7.21023E-10	2.95463E-06	0.000182449
Human papillomavirus infection	9.46109E-08	4.17865E-06	4.96167E-05	0.00152346
PI3K/Akt signaling pathway	0.00054035	0.004646518	0.00015513	0.003326039
JAK/STAT signaling pathway	0.003727101	0.016812323	0.000161589	0.003326039
MAPK/ERK signaling pathway	2.34192E-06	6.20608E-05	0.000655807	0.012460331
Pathways in cancer	7.73424E-10	6.83191E-08	0.000800177	0.014117417
Influenza A	0.001568909	0.010660536	0.001599311	0.023237052
Measles	0.011288239	0.034383717	0.00346524	0.039814879

RGCs, retinal ganglion cells; CNTF, ciliary neurotrophic factor; KEGG, Kyoto Encyclopedia of Genes and Genomes; FDR, false discovery rate.

### RNA Sequencing

**Figure 6.**
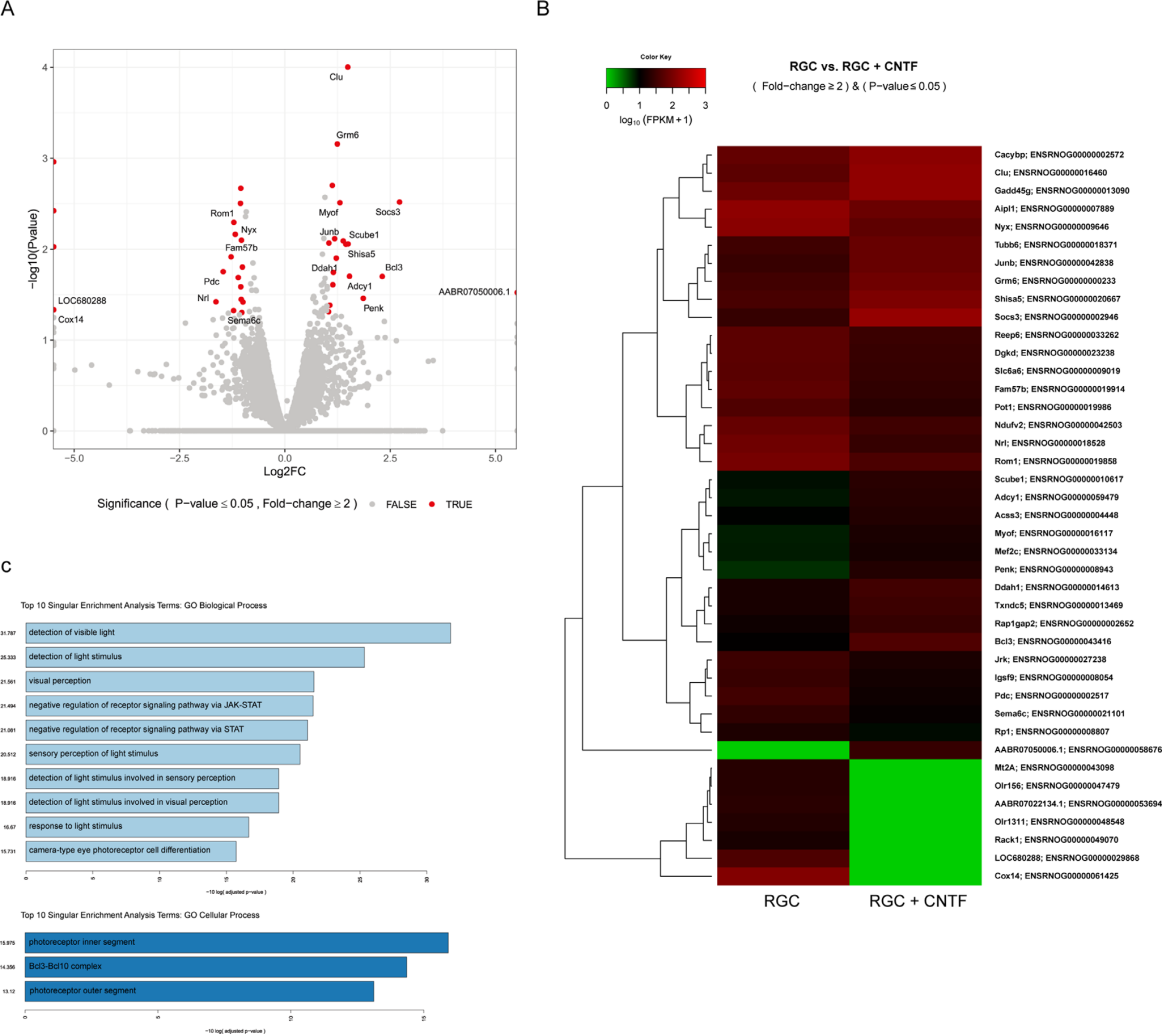
RNA sequencing revealed differential gene expression and pathway analysis revealed several upregulated and downregulated pathways in ciliary neurotrophic factor (CNTF)–treated retinal ganglion cells (RGCs). (**A**) Volcano plot showing log2-fold change in gene expression after CNTF treatment. Each dot represents a single gene. Totally, 41 genes (19 upregulated and 22 downregulated) were identified using differential gene expression analysis (>2-fold change and adjusted *P* value < 0.05). (**B**) Heat map of gene expression in RGCs. (**C**) Gene Ontology (GO) biological process and cellular process analyses. Bar size indicates the level of significance for each pathway (i.e., −log[P value]).

To elucidate gene expression differences and the specific cellular pathways affected by CNTF treatment, RNA sequencing was conducted. After stabilizing the isolated RGCs for 24 hours, they were supplemented with 50 ng/mL of CNTF and incubated for 24 hours, and then RNA sequencing was performed. Initial analysis revealed significant alterations in the gene expression profile of RGCs owing to CNTF treatment. A total of 41 genes (19 upregulated and 22 downregulated) showed >2-fold change with an adjusted *P* value <0.05 (Fig. 6). Table 4 shows the list of up- and downregulated genes and their biological processes. Of the upregulated genes, several genes were associated with the intrinsic apoptotic signaling pathway in response to DNA damage, negative regulation of receptor signaling pathway via JAK/STAT, and negative regulation of receptor signaling pathway via STAT. The downregulated genes included those involved in camera-type eye photoreceptor cell differentiation. Few up- and downregulated genes were associated with detection of light stimulus, detection of light stimulus involved in sensory perception, detection of light stimulus involved in visual perception, detection of visible light, response to light stimulus, sensory perception of light stimulus, and visual perception.

**Table 4.** Genes Identified as Upregulated or Downregulated Through RNA Sequencing and Associated Biological Processes

Official Gene Symbol	log2 (Fold Change)	*P* Value	Full Description of the Gene	Gene Ontology Biological Process
Upregulated				
*Acss3*	1.0535	0.042	acyl-CoA synthetase short-chain family member 3	
*Bcl3*	2.2983	0.020	BCL3, transcription coactivator	Intrinsic apoptotic signaling pathway in response to DNA damage, negative regulation of receptor signaling pathway via JAK-STAT, negative regulation of receptor signaling pathway via STAT, response to light stimulus
*Clu*	1.4798	<0.001	Clusterin	Intrinsic apoptotic signaling pathway in response to DNA damage
*Grm6*	1.2321	0.001	Glutamate metabotropic receptor 6	Detection of light stimulus, detection of light stimulus involved in sensory perception, detection of light stimulus involved in visual perception, detection of visible light, response to light stimulus, sensory perception of light stimulus, visual perception
*Junb*	1.3722	0.008	JunB proto-oncogene, AP-1 transcription factor subunit	
*Scube1*	1.4343	0.009	Signal peptide, CUB domain and EGF like domain containing 1	
*Shisa5*	1.4861	0.009	shisa family member 5	Intrinsic apoptotic signaling pathway in response to DNA damage
*Socs3*	2.7084	0.003	Suppressor of cytokine signaling 3	Negative regulation of receptor signaling pathway via JAK-STAT, negative regulation of receptor signaling pathway via STAT
Downregulated				
*Aipl1*	−1.059	0.002	Aryl hydrocarbon receptor-interacting protein-like 1	Detection of light stimulus, detection of visible light, response to light stimulus, sensory perception of light stimulus, visual perception
*Dgkd*	−1.067	0.003	Diacylglycerol kinase, delta	
*Fam57b*	−1.29	0.012	TLC domain containing 3B	
*Igsf9*	−1.059	0.026	Immunoglobulin superfamily, member 9	
*Nrl*	−1.649	0.038	Neural retina leucine zipper	Camera-type eye photoreceptor cell differentiation
*Pdc*	−1.477	0.018	Phosducin	
*Rom1*	−1.225	0.005	Retinal outer segment membrane protein 1	Camera-type eye photoreceptor cell differentiation, detection of light stimulus, detection of light stimulus involved in sensory perception, detection of light stimulus involved in visual perception, detection of visible light, response to light stimulus, sensory perception of light stimulus, visual perception
*Sema6c*	−1.23	0.048	Semaphorin 6C	
*Slc6a6*	−1.048	0.036	Solute carrier family 6 member 6	

Genes that showed infinite fold change or did not show consistent results between three experimental samples were excluded.

## Discussion

In this study, we observed protective effects of astrocytes on RGCs, especially in terms of viability and the expression of β3-tubulin. In addition, astrocytes also increased the percentage of viable RGCs under oxidative stress. CNTF seems to play a crucial role in these effects of astrocytes. According to the results of analysis using pathway inhibitors, the PI3K/AKT pathway was associated with both the viability and β3-tubulin expression of RGCs. On the contrary, the JAK/STAT pathway was related to the viability of RGCs and percentage of viable RGCs, and the MAPK/ERK pathway was only related to β3-tubulin expression. RNA sequencing results revealed CNTF-upregulated genes associated with the response to DNA damage and the JAK/STAT signaling pathway as well as CNTF-downregulated genes associated with photoreceptor cell differentiation.

Astrocytes are known to have various roles in the CNS. They form close interactions with neurons to provide structural support and engage in metabolic coupling; additionally, they serve as a nutrient source and storage for neurons.^32^ For many decades, it has been believed that astrogliosis and the formation of a glial scar inhibit axonal re-growth and plays a detrimental role in the neurological outcome. However, an increasing amount of evidence suggests that astrocytes also beneficial for neurons.^33^^,^^34^ Studies have demonstrated that astrocytes respond differentially to varying types of CNS insults.^35^^–^^38^ A1- and A2-reactive astrocytes are known to be induced by neuroinflammation and ischemia, respectively. A1-reactive astrocytes upregulate the expression of numerous genes, including complement cascade genes, leading to damage synapses, thus performing “harmful” functions. The ischemia-induced A2-reactive astrocytes, on the contrary, enhance synaptic repair by upregulating the expression of several neurotrophic factors, thereby promoting neuronal survival and growth.^39^ In this study, oxidative stress was used to trigger apoptosis. Oxidative stress may convert the astrocytes into A2-responsive cells, demonstrating a protective role for RGCs as well as ischemia.

The protective effects of astrocytes was comparable to those of Müller cells, according to the findings of this study. In certain experiments, astrocytes exhibited a better protective effect than Müller cells. Müller cells, similar to astrocytes, serve a number of functions. They can supply neurotrophic factors and antioxidants to RGCs in order to maintain viability in RGCs.^40^^,^^41^ Several studies have shown that astrocytes synthesize and release CNTF, whereas Müller cells are a major source of BDNF in addition to NGF and GDNF.^10^^,^^42^^,^^43^ These findings are in agreement with our findings, which show that CNTF plays an essential role in the effects of astrocytes. One previous study compared the effects of astrocytes and Müller cells on RGCs. Although both cells protect RGCs, astrocytes may be more advantageous for RGC neurite development than Müller cells.^44^ The impacts of astrocytes and Müller cells on RGCs may vary depending on the developmental stage of RGCs. We did not perform any further experiments on this because comparing the two cells was not the primary purpose of this study. Further research is warranted to investigate the differences in the actions of the two glial cells in the same environment.

We used a membrane insert to prevent direct contact between astrocytes and RGCs, allowing only soluble factors to move between the two cells. Our findings showed that soluble factors originating from astrocytes improved RGC viability and expression of β3-tubulin and increased the number of viable RGCs under oxidative stress. CNTF is a strong candidate for a soluble factor, based on the results of the ELISA and anti-CNTF treatment. Furthermore, RT-PCR findings suggested that astrocytes are likely the source of increased synthesis of CNTF. One of the unexpected findings was that CNTF synthesis was increased when astrocytes were incubated with RGCs. This result suggested that RGCs released a soluble factor that stimulated astrocytes to synthesize CNTF. Various triggers have been reported to stimulate CNTF release from astrocytes, including lens injury, intravitreal zymosan application, and inflammatory stimulation.^10^^,^^45^^,^^46^ In contrast, one study found that disrupting the contact between neurons and astrocytes enhanced CNTF production in astrocytes and concluded that diffusive molecules had little effect on CNTF production.^47^ However, based on our findings, we suspect that the loss of direct interaction between RGCs and astrocytes does not increase CNTF synthesis in astrocytes directly, but CNTF production is promoted by a substance secreted by RGCs as a result of loss of contact.

CNTF treatment alone also improved the viability of and β3-tubulin expression of RGCs and increased the percentage of viable RGCs under oxidative stress. Several studies have reported the neuroprotective effects of CNTF. CNTF promoted the survival of purified RGCs in vitro^48^ and in an optic nerve axotomy animal model.^49^ Furthermore, CNTF plays a role in axogenesis. Purified rat RGCs in serum-free medium containing CNTF showed extensive long neurite outgrowth.^50^^,^^51^ In addition, intravitreal CNTF injections enhanced RGC axonal regeneration into peripheral nerve grafts after axotomy.^52^ One interesting finding of our study was that a specific concentration of CNTF showed the maximal protective effect. Another previous study reported that CNTF stimulated axonal regeneration in RGCs in a dose-dependent manner.^53^ However, our results showed that the protective effect of CNTF was most pronounced at a concentration of 50 ng/mL and that the effect was not significantly increased as the concentration of CNTF increased above 50 ng/mL. Previous studies reporting a dose-dependent effect of CNTF used 2 to 3-month-old adult mice,^53^ whereas our study used 2 to 3-day-old rats. Thus, this discrepancy suggests that the effects of CNTF do not always occur in a dose-dependent manner. Another study using adipose-derived mesenchymal stem cells found that low concentrations of CNTF (5 ng/mL) promoted cell proliferation, whereas higher doses (50 and 100 ng/mL) inhibited it.^54^ These findings imply that CNTF shows the greatest protective effect on RGCs at a specific concentration, which varies depending on the cell type and developmental stage.

Furthermore, through microarray analysis, we identified the JAK/STAT, MAPK/ERK, and PI3K/AKT pathways as the pathways that were altered in RGCs as a response to CNTF treatment and in a co-culture with astrocytes. The functional effects of each pathway were then determined using pathway inhibitors. CNTF effects on RGC viability were decreased by both JAK inhibitor AG490 and PI3K inhibitor LY294002. CNTF increased the percentage of viable RGCs under oxidative stress; this was also decreased by the JAK inhibitor AG490. β3-tubulin expression was reduced by the MAPK/ERK pathway inhibitor PD98059 and the JAK inhibitor AG490. Exogenous CNTF has been shown to increase neuronal survival and axonal regeneration by activating the MAPK/ERK, PI3K/AKT, and JAK/STAT pathways.^55^ In addition, blockade of signaling pathways using LY294002, PD98059, and AG490 inhibited the CNTF/CPT-cAMP-dependent survival and regeneration effects in RGCs.^56^ Another study found that adding CNTF to adult RGC culture did not affect survival but stimulated neurite outgrowth via the JAK/STAT and PI3K/AKT signaling pathways.^53^ This disparity could be attributed to the RGC maturation status. Enhanced RGC survival was observed in a study using relatively young (8–10-week-old) rats, as well as in our experiment using neonatal rats, whereas a previous study in adult rats found improvements only in neurite length but not RGC survival. This result suggests that the beneficial effects of CNTF on RGCs may be limited with age. Moreover, the relevance of each pathway can be influenced not just by RGC maturation but also by the environment in which the RGCs are located. In a previous study, suppression of the PI3K/AKT, JAK/STAT, and MAPK/ERK pathways increased RGC survival following optic nerve axotomy, opposing our findings.^24^ The same study also showed that inhibition of the PI3K/AKT or JAK/STAT pathway protects RGCs via an activated macrophage mechanism, whereas the MAPK/ERK route does so via a macrophage-independent mechanism.^24^ The absence or presence of macrophages might explain these contradictory findings. In other words, the impact of signaling pathways, including PI3K/AKT, JAK/STAT, and MAPK/ERK pathways, on RGCs may vary depending on the interactions between RGCs and surrounding cells. Thus, additional research is needed to identify how the effects of signaling pathways on RGCs are affected by RGC maturity and the cells that interact with them.

RNA sequencing was applied to reveal the genes up- and downregulated by CNTF treatment. One of the upregulated genes, *SOCS3*, has been reported to be associated with the proliferation of neural tissue.^57^ Expression of clusterin, a stress-related gene that promotes cell survival,^58^ was also increased. Additionally, several upregulated genes, such as *shisa5* and *BCL3*, were identified, and their biological processes were reported but their relationships with RGCs were not. Interestingly, CNTF-downregulated genes included those involved in photoreceptor cell differentiation, such as *Nrl* and *Rom1*.^59^^,^^60^ Because RGCs were harvested from 2- to 3-day-old rats in our study, RGCs may still exhibit some of the characteristics of retinal progenitor cells. This study speculates that CNTF can affect retinal progenitor cells in the early stage of differentiation to inhibit differentiation into photoreceptor cells and promote differentiation into RGCs. In addition, several genes were identified that have not been reported for their association with RGCs and their biological processes. The role of each gene identified in this study will need to be elucidated in further studies.

This study had several limitations. First, we used neonatal rat RGCs rather than adult RGCs; the vulnerability of neonatal RGCs to apoptotic stimuli may differ from that of adult RGCs.^61^ However, as previously reported,^62^^,^^63^ neonatal RGCs share many characteristics with adult RGCs; therefore, they could provide substantial insights regarding the survival and development of RGCs. Second, because the current study was conducted only in vitro, the results may differ from those observed in vivo, where RGCs interact with many other cells such as Müller cells and other glial cells. It is necessary to confirm whether the outcomes of this study can be reproduced in vivo. Third, RGCs were incubated for a total of 2 days in this investigation: 1 day for post-harvest stability and 1 day after the intervention (astrocyte co-culture or CNTF treatment). In comparison to the entire developmental period of the RGCs, this was a short period. As a result, the evaluation of neurite growth was limited, and further research examining RGC neurite growth over a longer period is required. Nonetheless, we believe our findings are significant because they show that astrocytes and CNTFs influence RGCs even when exposed for short periods of time. Fourth, when interpreting the ATP assay results, there may be concerns that these results reflect the metabolic activity rather than viability itself. The ATP assay is known to be the fastest cell viability assay and tends to produce less artifacts than other viability assay methods,^64^ and thus, we have used this method in previous studies.^25^^,^^62^ However, as the increased luminescence may be due to increased metabolic activity within viable RGCs, we also performed flow cytometry to provide quantitative results. In spite of these limitations, this study has strengths in that the data indicate that the protective effects of astrocytes on RGCs are comparable to those of Mueller cells and demonstrate the role of each signaling pathway, including JAK/STAT, PI3K/AKT, and MAPK/ERK pathways. RNA sequencing was applied to analyze the genes that were up- and downregulated following CNTF treatment, which was another advantage of this study.

In conclusion, our results show that astrocytes play protective roles for RGCs and that CNTF plays a crucial role in the process. We also found that CNTF influenced RGCs via the JAK/STAT, MAPK/ERK, and PI3K/AKT pathways. Finally, RNA sequencing analysis revealed that CNTF-upregulated genes were involved in the response to DNA damage and -downregulated genes were involved in photoreceptor differentiation. The findings of this study have important implications for using CNTF and astrocytes as therapeutic agents for neurodegenerative diseases, such as glaucoma.

## Supplementary Material

Supplement 1
