# Supplementary material for: Ciliary Neurotrophic Factor Derived From Astrocytes Protects Retinal Ganglion Cells Through PI3K/AKT, JAK/STAT, and MAPK/ERK Pathways
Source: Invest Ophthalmol Vis Sci. 2022 Aug 4;63(9):4. doi: 10.1167/iovs.63.9.4 (PMC9363680; doi:10.1167/iovs.63.9.4)
Supplement: Supplement 1 [file iovs-63-9-4_s001.pdf]

Supplementary materials

Figures

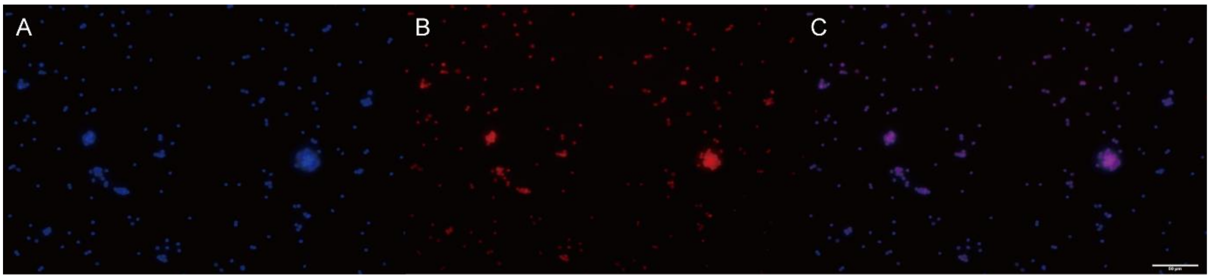

Supplemental Figure 1. Immunofluorescence of primary retinal ganglion cells (RGCs) at day 2 of culture shows positive expression of BRn3a (B). DAPI nuclear staining is shown in (A) and in the merged image in (C). Scale bar, 50 μm.

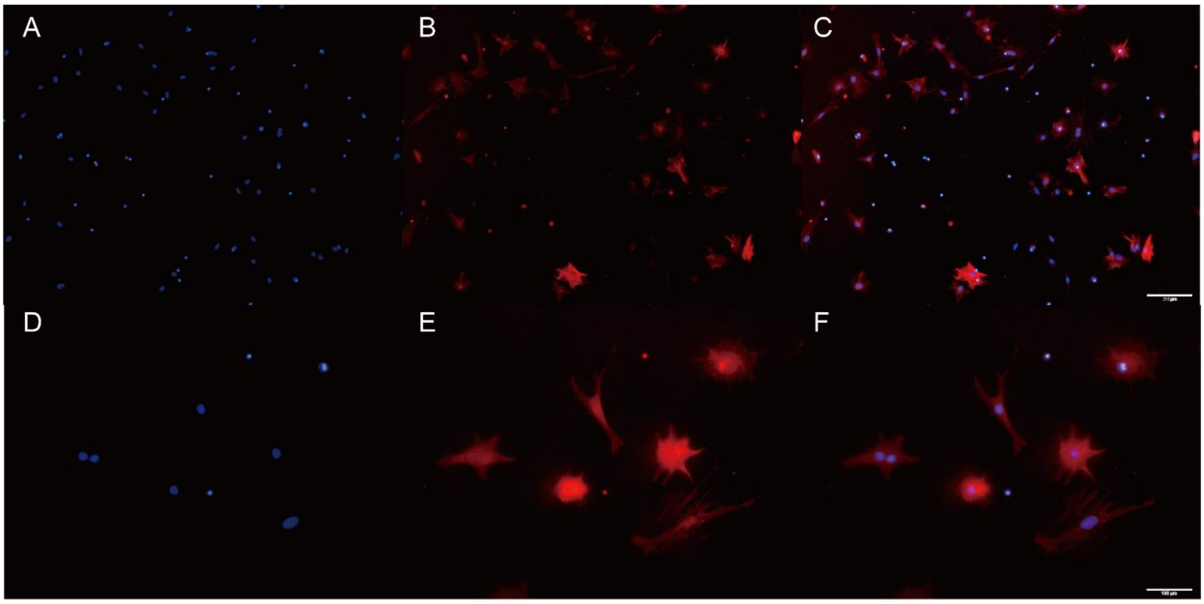

Supplemental Figure 2. Immunofluorescence of astrocytes at day 2 of culture shows positive expression of GFAP (B and E). DAPI nuclear staining is shown in (A and D) and in the merged images in (C and F). A-C: Scale bar, 250 μm; D-F: Scale bar, 100 μm.

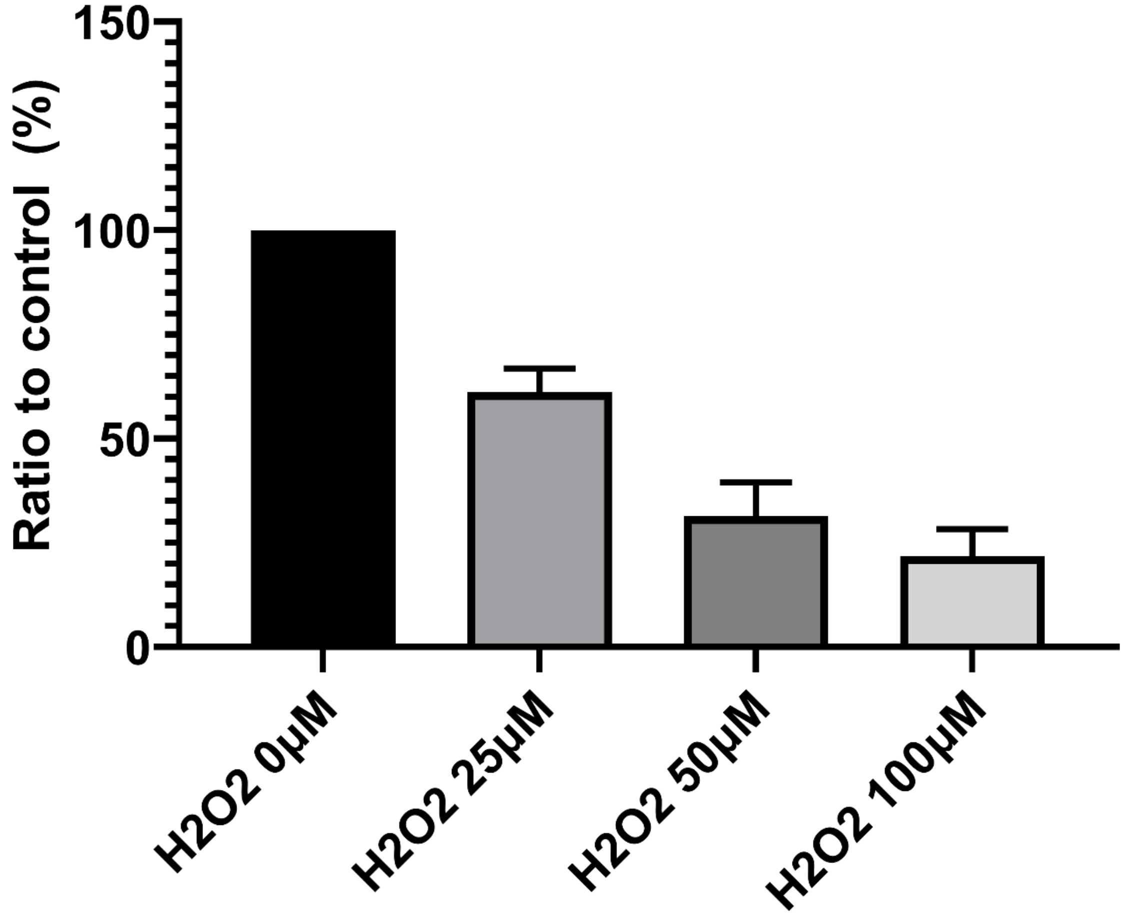

Supplemental Figure 3. Results of cell counting. Primary mouse retinal ganglion cells (RGCs) were exposed to oxidative stress induced by hydrogen peroxide (H_2O_2) for up to 24 hours. The number of DAPI stained-RGCs was decreased in a dose-dependent manner. Data in the columns indicate the mean ± SD of three experiments.
